# A 7-Deazaadenosylaziridine Cofactor for Sequence-Specific Labeling of DNA by the DNA Cytosine-C5 Methyltransferase M.HhaI

**DOI:** 10.3390/molecules201119723

**Published:** 2015-11-23

**Authors:** Falk Kunkel, Rudi Lurz, Elmar Weinhold

**Affiliations:** 1 Institute of Organic Chemistry, RWTH Aachen University, Landoltweg 1, Aachen D-52056, Germany; Falk_Schmidt@gmx.de; 2 Max-Planck-Institute for Molecular Genetics, Ihnestrasse 73, Berlin D-14195, Germany; Lurz@molgen.mpg.de

**Keywords:** *S*-adenosyl-l-methionine, AdoMet, aziridine, CpG methylation, cofactor, 7-deazaadenosine derivative, DNA methyltransferase, DNA methylation, DNA labeling, Sonogashira coupling, SAM

## Abstract

DNA methyltransferases (MTases) catalyze the transfer of the activated methyl group of the cofactor *S*-adenosyl-l-methionine (AdoMet or SAM) to the exocyclic amino groups of adenine or cytosine or the C5 ring atom of cytosine within specific DNA sequences. The DNA adenine-N6 MTase from *Thermus aquaticus* (M.TaqI) is also capable of coupling synthetic *N*-adenosylaziridine cofactor analogues to its target adenine within the double-stranded 5′-TCG**A**-3′ sequence. This M.TaqI-mediated coupling reaction was exploited to sequence-specifically deliver fluorophores and biotin to DNA using *N*-adenosylaziridine derivatives carrying reporter groups at the 8-position of the adenine ring. However, these 8-modified aziridine cofactors were poor substrates for the DNA cytosine-C5 MTase from *Haemophilus haemolyticus* (M.HhaI). Based on the crystal structure of M.HhaI in complex with a duplex oligodeoxynucleotide and the cofactor product, we synthesized a stable 7-deazaadenosylaziridine derivative with a biotin group attached to the 7-position via a flexible linker. This 7-modified aziridine cofactor can be efficiently used by M.HhaI for the direct, quantitative and sequence-specific delivery of biotin to the second cytosine within 5′-G**C**GC-3′ sequences in short duplex oligodeoxynucleotides and plasmid DNA. In addition, we demonstrate that biotinylation by M.HhaI depends on the methylation status of the target cytosine and, thus, could provide a method for cytosine-C5 DNA methylation detection in mammalian DNA.

## 1. Introduction

Deoxyribonucleic acid (DNA) carries the genetic information of all living organisms, and the information is stored in the sequence of the four nucleobases adenine, cytosine, guanine and thymine. In most organisms, the information content of DNA is increased by enzymatic methylation of the exocyclic amino groups of adenine or cytosine or the C5-ring atom of cytosine within short DNA sequences (epigenetic information). All three types of DNA methylation are found in bacteria and serve a broad range of biological processes, including protection against endogenous restriction endonucleases, direction of DNA mismatches repair after DNA replication and regulation of gene expression. In mammalian cells, DNA methylation is restricted to cytosine-C5 within 5′-CG-3′ (CpG) sequences and involved in transcriptional regulation of gene expression and cell differentiation.

**Scheme 1:**
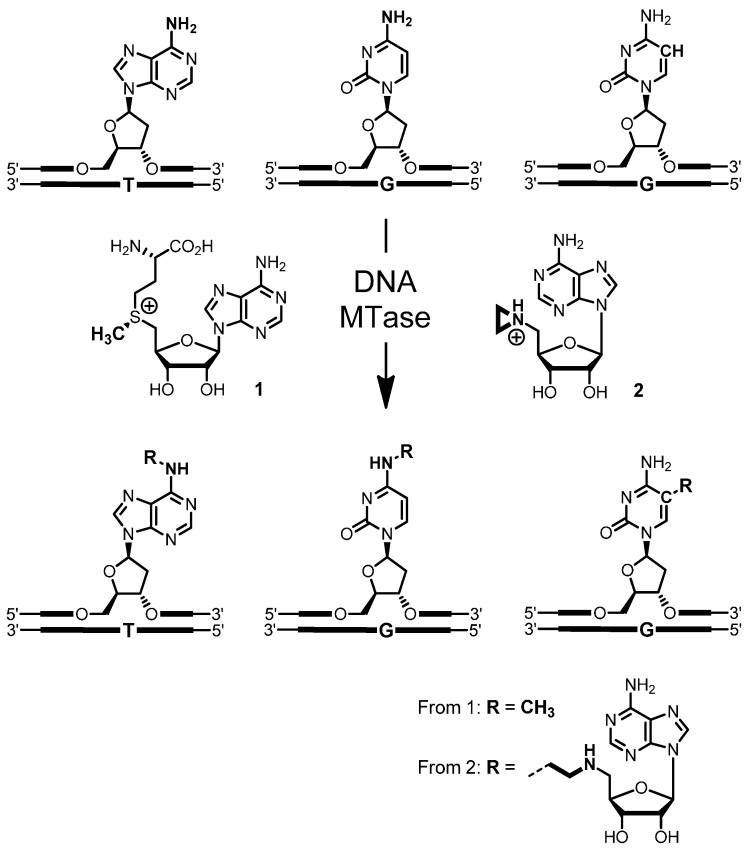
Reactions catalyzed by the three classes of DNA methyltransferases (MTase). (**Left**) Natural methyl group transfer from the cofactor AdoMet **1** leads to methylated nucleobases within specific DNA sequences (black bars); (**Right**) the synthetic aziridine cofactor **2** is sequence-specifically coupled with the target base.

**Scheme 2:**
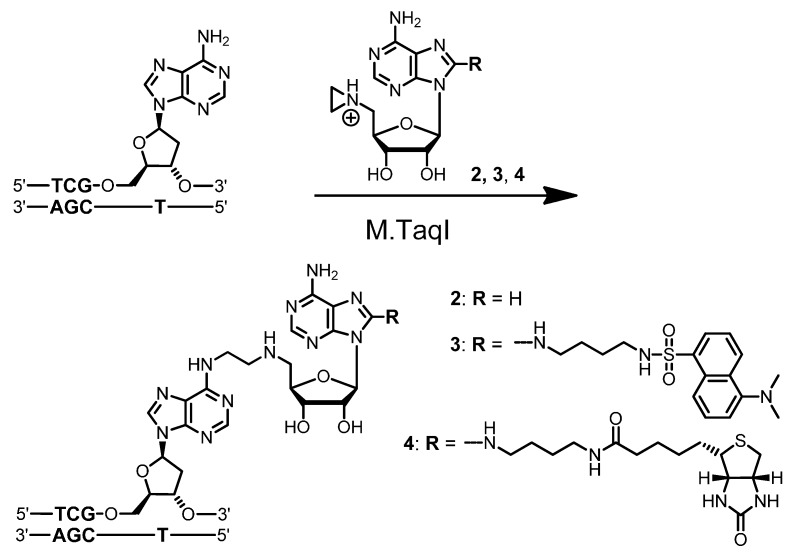
Coupling of *N*-adenosylaziridine (**2**) or the 8-modified *N*-adenosylaziridine cofactors **3** and **4** by the DNA MTase from *Thermus aquaticus* (M.TaqI) for sequence-specific labeling of DNA with the dansyl fluorophore or biotin.

DNA methylation is catalyzed by DNA methyltransferases (MTases), which transfer the activated methyl group from the cofactor *S*-adenosyl-l-methionine (AdoMet or SAM, **1**) to adenine-N6, cytosine-N4 or cytosine-C5 within specific double-stranded DNA sequences ranging from two to eight base pairs (Scheme 1, left) [1]. These DNA MTases can be regarded as tools for sequence-specific covalent modification of DNA. However, the transferred methyl group is a limited reporter group and only suitable for isotope labeling, including radioactive isotopes, like tritium or carbon-14. This limitation can be overcome with synthetic cofactor analogues. *N*-adenosylaziridine (**2**), for example, functions as a cofactor for the representatives of all three classes of DNA MTases and is coupled with the target nucleobases by nucleophilic aziridine ring opening (Scheme 1, right). The aziridine cofactor **2** is quantitatively, base- and sequence-specifically coupled with its target adenine within the sequence 5′-TCG**A**-3′ (target adenine in bold) by the DNA adenine-N6 MTase from *Thermus aquaticus* (M.TaqI) [2] and can be used as a transfer reagent for reporter groups, like fluorophores and biotin. Based on the crystal structure of M.TaqI in complex with a short duplex oligodeoxynucleotide (ODN) and a cofactor analogue [3], *N*-adenosylaziridine derivatives were synthesized carrying either a dansyl fluorophore **3** or a biotin group **4** at the 8-position of the adenine ring [4,5,6,7]. These 8-modified *N*-adenosylaziridine derivatives are coupled with DNA by M.TaqI resulting in sequence-specifically labeled 5′-TCGA-3′ sequences in various DNA substrates (Scheme 2). This enzymatic method for DNA labeling was named “sequence-specific methyltransferase-induced labeling of DNA” (SMILing DNA) [5]. Other groups used the SMILing technique in combination with additional DNA MTases for DNA labeling [8,9,10,11,12,13]. However, labeling was performed in two-steps, *i.e.*, enzymatic attachment of a unique functional to DNA followed by chemo-selective chemical modification. Although such a two-step procedure offers the flexibility of using one aziridine cofactor for labeling with different reporter groups, it adds an extra chemical step, which is difficult to quantify on the DNA and can lead to partial DNA labeling.

The prokaryotic DNA cyotsine-C5 MTase from *Haemophilus haemolyticus* (M.HhaI) is of special interest, because it methylates the second cytosine within the sequence 5′-G**C**GC-3′ (target cytosine in bold) which includes the 5′-CG-3′ recognition sequence of mammalian DNA MTases [14]. Thus, M.HhaI could serve as useful tool for studying mammalian DNA methylation by SMILing DNA. Unfortunately, the 8-modified *N*-adenosylaziridine derivatives **3** and **4** are poor substrates for M.HhaI. To overcome this limitation, we report here the design and synthesis of a 7-modified aziridine cofactor, which is suitable for quantitative labeling with M.HhaI in one step and for CpG-methylation detection.

## 2. Results and Discussion

### 2.1. Design and Synthesis of an N-Adenosylaziridine Cofactor for DNA Labeling with M.HhaI

**Figure 1:**
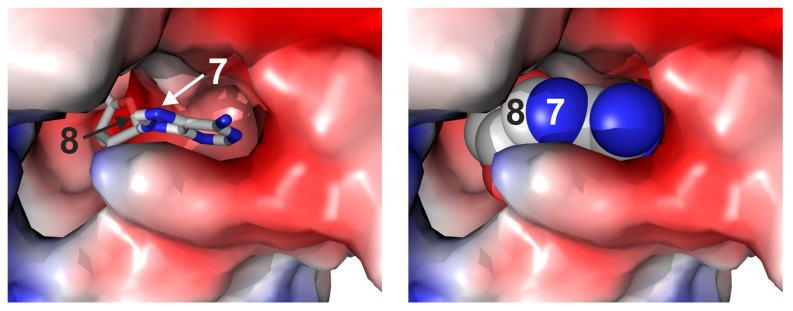
Cofactor binding pocket of the DNA MTase from *Haemophilus haemolyticus* (M.HhaI) (Protein Data Bank Entry Number 1MHT) shown as an electrostatic surface potential structure (red denotes negative potential, blue positive potential and white neutral). The bound demethylated cofactor product *S*-adenosyl-l-homocysteine (AdoHcy) is presented as a stick model (**left**) and as a space-filling model (**right**). The 7-position of the adenine ring is pointing towards the solvent, while the 8-position is partially blocked by the protein.

Although M.HhaI and M.TaqI can utilize *N*-adenosylaziridine (**2**) equally well, M.HhaI in contrast to M.TaqI cannot efficiently couple the 8-modified *N*-adenosylaziridine derivatives **3** and **4** with DNA (*vide infra*). This result is in agreement with the crystal structure of M.HhaI in complex with a duplex ODN and *S*-adenosyl-l-homocysteine (AdoHcy), the demethylated cofactor formed after methyl group transfer from the natural cofactor AdoMet **1** [15]. AdoHcy is buried in the cofactor binding pocket of M.HhaI and the 8-position of the adenine points towards the enzyme (Figure 1). Thus, attaching groups to this position could lead to sterically unfavorable interactions and loss of binding affinity. In contrast, the 7-position of the adenine ring is pointing towards the solvent, and modifications at this position should not interfere with cofactor binding. However, alkylation of the N7 atom will formally place a positive charge onto the adenine ring, which will weaken the glycosidic bond and enhance depurination. This problem can be overcome by replacing nitrogen with carbon and synthesizing the stable 7-deazaadenosylaziridine derivative **5** with biotin attached via a flexible linker to the 7-position.

**Scheme 3:**
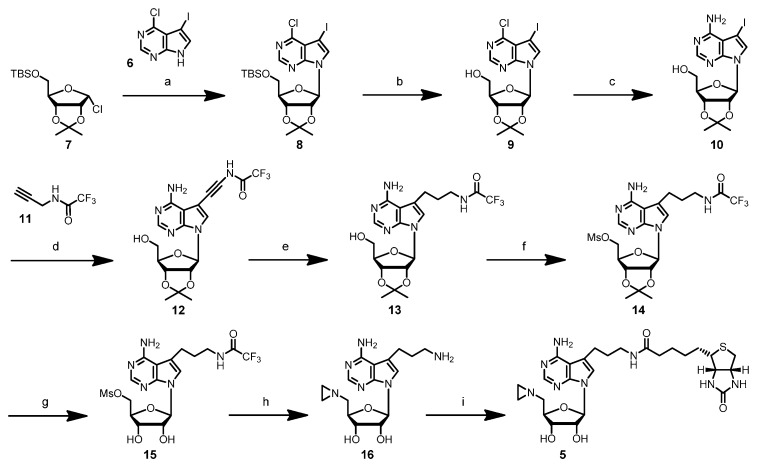
Synthesis of the 7-modified 7-deazaadenosylaziridine cofactor 5. (**a**) KOH, tris[2-(2-methoxyethoxy)ethyl]amine (TDA-1), 43%. (**b**) tetrabutylammonium fluoride (TBAF), 69%. (**c**) MeOH/NH_3_, 59%. (**d**) Pd(PPh_3_)_4_, CuI, NEt_3_, 98%. (**e**) PtO_2_, H_2_, 81%. (**f**) MsCl, DMAP, NEt_3_, 46%. (**g**) TFA. (**h**) (1) Aziridine, *N*-ethyldiisopropylamine (EDIA), (2) NHEt_3_HCO_3_, 39% (for g and h). (**i**) *N*-hydroxysuccinimidyl (NHS) biotin, 32%.

The synthesis of the 7-modified aziridine cofactor **5** (Scheme 3) started by coupling 4-chloro-5-iodo-7*H*-pyrrolo[2,3-*d*]pyrimidine (**6**) with 5-*O*-(*t*-butyldimethylsilyl)-2,3-*O*-isopropylidene-*α*-d-ribofuranosyl chloride (**7**). Initial attempts to deprotonate **6** with sodium hydride according to Ugarkar *et al.* [16] gave only small amounts of product **8**. We, therefore, adapted the method by Rosemeyer and Seela [17], which employs potassium hydroxide in combination with the cryptand tris[2-(2-methoxyethoxy)ethyl]amine (TDA-1) and obtained nucleoside **8** in an acceptable yield. After removal of the TBS protecting group with tetrabutylammonium fluoride, the chlorine atom in nucleoside **9** was substituted in saturated methanolic ammonia under elevated pressure to yield the 7-deazaadenosine derivative **10**. Removal of the TBS protecting group at this stage of the synthesis was advantageous, because the TBS group was partially cleaved off by the ammonia treatment, leading to a product mixture. The side chain at the 7-position was introduced by palladium-catalyzed Sonogashira coupling with *N*-(2-propynyl)-2,2,2-trifluoroacetamide (**11**) in analogy to a procedure by Seela *et al.* [18], and the alkyne linker of **12** was reduced to the more flexible alkyl linker by catalytic hydrogenation. The next steps followed our synthesis of the 8-modified aziridine cofactor **4** [7]. The 5′-hydroxy group was activated as a mesyl ester and the isopropylidene protecting group removed under acidic conditions. The mesylate was replaced with aziridine in a nucleophilic substitution reaction, the trifluoroacetyl protecting group removed under basic aqueous work up and the resulting primary amine coupled with *N*-hydroxysuccinimidyl biotin (NHS biotin) to yield the desired 7-deazaadenosylaziridine derivative **5** with biotin attached to the 7-position.

### 2.2. Labeling Short Duplex Oligodeoxynucleotides with M.HhaI and ***5***

**Scheme 4:**
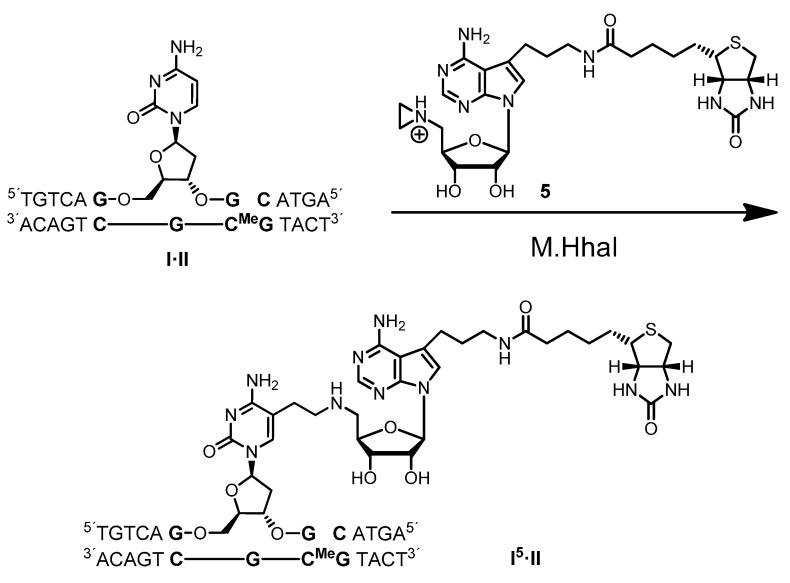
Coupling of the 7-modified aziridine cofactor **5** with the duplex oligodeoxynucleotide (ODN) **I·II** by the DNA MTase M.HhaI. The recognition sequence of M.HhaI is printed in bold face (C^Me^ = *C*5-methylcytosine).

To test whether the new 7-deazaadenosylaziridine derivative **5** functions as a cofactor for M.HhaI (Scheme 4), we initially used the short duplex ODN **I·II** (13 base pairs). Duplex **I·II** with the palindromic 5′-GCGC-3′ recognition sequence was prepared by annealing Strand **I** with the complementary Strand **II**. The target cytosine in the lower Strand **II** was blocked by methylation, *i.e.*, cytosine is replaced with *C*5-methylcytosine (C^Me^), and only the target cytosine in the upper Strand **I** can be modified by M.HhaI. Formation of Duplex **I·II** was analyzed by anion-exchange HPLC, and the duplex eluted as a single peak after 18.4 min.

**Figure 2:**
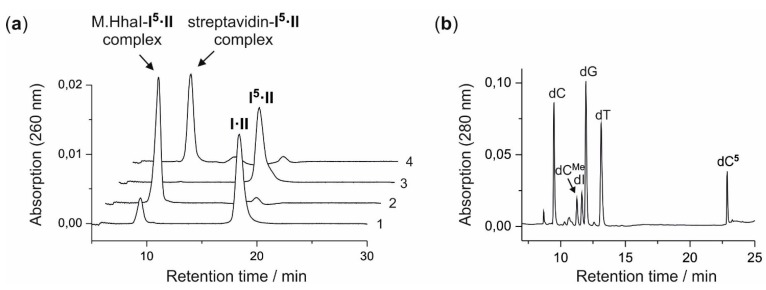
Biotin labeling of duplex ODN **I·II** with aziridine cofactor **5** and M.HhaI. (**a**) Anion-exchange HPLC analysis of the coupling reaction between the aziridine cofactor **5** and the duplex **I·II** by M.HhaI. (Trace 1) Analysis directly after mixing the components; (Trace 2) analysis after 3 h of incubation at 37 °C; (Trace 3) analysis after 3 h of incubation at 37 °C followed by the release of the modified duplex **I^5^·II** from the protein-DNA complex by heat denaturation; (Trace 4) analysis of streptavidin binding to **I^5^·II**; (**b**) Enzymatic fragmentation of the product duplex **I^5^·II** with DNase I, phosphodiesterase from *Crotalus adamanteus*, phosphodiesterase from calf spleen and alkaline phosphatase analyzed by reverse-phase HPLC. The modified nucleoside (dC**^5^**) elutes after 2’-deoxycytidine (dC), *C*5-methyl-2’-deoxycytidine (dC^Me^), 2’-deoxyinosine (dI), 2’-deoxyguanosine (dG) and 2’-deoxythymidine (dT) (2’-deoxyadenosinewas converted to dI by contaminating adenosine deaminase activity during the fragmentation reaction).

Duplex **I·II** was incubated with M.HhaI and 7-deazaadenosylaziridine derivative **5**, and the coupling reaction was followed by anion exchange HPLC (Figure 2). Directly after mixing of all components, the formation of a new compound with a retention time of 9.8 min was observed (Figure 2a, Trace 1). In absence of either M.HhaI or **5**, no formation of any additional compound was detected, indicating that the formation requires both M.HhaI and **5**. Based on the altered UV-absorption ratio (260 nm/280 nm), the new compound was assigned to be a protein-DNA complex consisting of the coupling product **I^5^·II** and M.HhaI (E_260/280_ (**I·II**) = 1.88; E_260/280_ (M.HhaI-**I^5^·II**) = 1.54). All duplex ODN **I·II** had reacted to the M.HhaI-**I^5^·II** complex after incubation at 37 °C for 3 h (Figure 2a, Trace 2). The protein-DNA complex between M.HhaI and the coupling product **I^5^·II** is very stable and does not dissociate during anion exchange chromatography. A similar behavior has been observed before for the coupling of 8-modified *N*-adenosylaziridine cofactor **3** with a short duplex ODN by the DNA MTase M.TaqI [4]. The sample was heated to 65 °C for 30 min to denature the protein-DNA complex, and the liberated product duplex **I^5^·II** (E_260/280_ = 1.80) eluted slightly earlier (17.7 min) than the starting duplex **I·II** (18.4 min) (Figure 2a, Trace 3). Residual cofactor **5** was removed by gel filtration and the presence of biotin in the coupling product **I^5^·II** verified by adding streptavidin. The formation of a new protein-DNA complex (E_260/280_ = 0.93) with a reduced retention time of 10.3 min is in agreement with the binding of streptavidin to the biotinylated duplex **I^5^·II** (Figure 2a, Trace 4).

The product duplex **I^5^·II** was characterized by enzymatic fragmentation with a mixture of DNase I, phosphodiesterase from *Crotalus adamanteus*, phosphodiesterase from calf spleen and alkaline phosphatase. The fragmentation products were analyzed by reverse-phase HPLC (Figure 2b). Beside the deoxynucleosides 2’-deoxycytidine (dC), *C*5-methyl-2’-deoxycytidine (dC^Me^), 2’-deoxyinosine (dI) (formed from 2’-deoxyadenosine by contaminating adenosine deaminase activity), 2’-deoxyguanosine (dG) and 2’-deoxythymidine (dT), a new compound eluting after 22.9 min was observed. This new compound was isolated and detected as a positively-charged ion at *m*/*z* 824.4 by electrospray ionization mass spectrometry (ESI-MS). The observed mass is in good agreement with the calculated mass (824.3) for 2′-deoxycytidine modified with **5** (dC**^5^**) and a sodium ion ([dC**^5^** + Na]^+^). Taken together, these results demonstrate that 7-deazaadenosylaziridine derivative **5** is a cofactor for M.HhaI and can be used to covalently attach biotin to short duplex ODN.

### 2.3. Biotin Labeling of pUC19 Plasmid DNA with M.HhaI and ***5***

**Figure 3:**
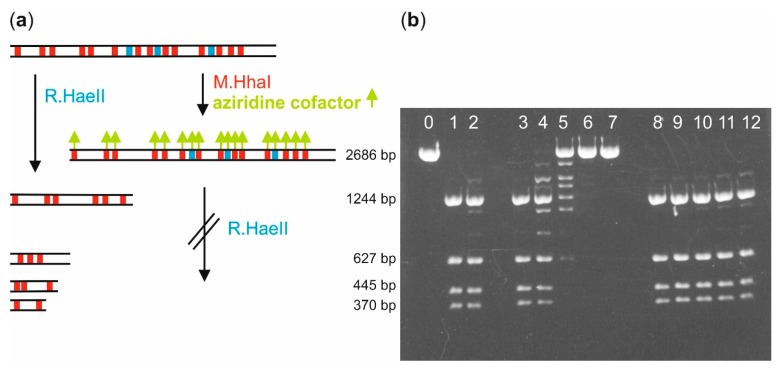
Biotin labeling of plasmid DNA with aziridine cofactor **5** and M.HhaI. (**a**) Schematic representation of the modification-protection assay used to demonstrate labeling of linearized pUC19 plasmid DNA (LpUC19). Coupling of an aziridine cofactor (green arrows) with the M.HhaI recognition sequences 5′-G**C**GC-3′ (red/blue boxes) by M.HhaI leads to protection of a subset of sites (blue boxes) against cleavage by R.HaeII restriction endonuclease (recognition sequence 5′-RGCGCY-3′, R = G or A, Y = C or T). Unmodified recognition sequences are cleaved by R.HaeII resulting in the formation of four DNA fragments on the agarose gel (1%). The DNA length is given in base pairs (bp); (**b**) Modification-protection assay for M.HhaI with 7-modified aziridine cofactor **5** (Lanes 4–7) and 8-modified aziridine cofactor **4** (9–12). Lane 0: LpUC19; Lane 1: LpUC19 cleaved by R.HaeII; Lane 2: LpUC19 incubated with M.HhaI alone for 60 min; Lane 3: LpUC19 incubated with **5** alone for 1 h; Lanes 4–7: LpUC19 incubated with M.HhaI and **5** for 0 min, 10 min, 30 min and 60 min, respectively; Lane 8: LpUC19 incubated with **4** alone for 60 min; Lanes 9–12: LpUC19 incubated with M.HhaI and **4** for 0 min, 10 min, 30 min and 60 min, respectively. Samples for Lanes 2–12 were treated with R.HaeII before agarose gel electrophoresis.

Next, we verified that M.HhaI and aziridine cofactor **5** can be used to label longer DNA fragments. pUC19 plasmid DNA (2686 bp) containing 17 recognition sequences for M.HhaI was selected as the test substrate. The pUC19 plasmid DNA was linearized by treatment with the restriction endonuclease R.XmnI (LpUC19) to facilitate analyses by agarose gel electrophoresis and electron microscopy (*vide infra*). To follow the labeling reaction, we employed a modification-restriction assay, which utilized the property of the restriction endonuclease R.HaeII to be unable to cleave its 5′-R**GCGC**Y-3′ recognition sequence (R = G or A, Y = C or T, overlapping M.HhaI recognition sequence in bold) when modified by M.HhaI [19]. LpUC19 contains three R.HaeII recognition sequences, and fragmentation by R.HaeII results four DNA fragments. Upon treatment of LpUC19 with M.HhaI and AdoMet **1** or another cofactor, the R.HaeII recognition sequences become modified, and R.HaeII is no longer capable of cleaving the modified LpUC19 plasmid DNA (Figure 3a).

Directly after mixing M.HhaI and the 7-modified aziridine cofactor **5** with LpUC19 plasmid DNA, some target sequences of R.HaeII were already blocked by coupling of **5** with M.HhaI recognition sequences (Figure 3b, Lane 4). After 30 min of incubation at 37 °C, the LpUC19 plasmid DNA was fully protected against cleavage by R.HaeII, indicating complete DNA modification (Figure 3b, Lane 6). Full protection was also observed when proteinase K was added after the modification reaction, and the plasmid purified before incubation with R.HaeII. This again indicates that the DNA becomes covalently modified. In contrast, complete fragmentation was observed in control experiments in the absence of either M.HhaI or cofactor **5**, which demonstrates that both DNA MTase and cofactor are required for DNA modification (Figure 3b, Lanes 2 and 3).

The importance of the attachment position for the label on the aziridine cofactor can be directly assessed from parallel experiments with M.HhaI and the 8-modified aziridine cofactor **4**. Nearly no protection against cleavage by R.HaeII was observed even after 1 h incubation, which shows that larger modifications at the 8-position of *N*-adenosylaziridine are not well tolerated by M.HhaI.

It should be noted that biotinylation of LpUC19 plasmid DNA with the 7-modified aziridine cofactor **5** was performed with a stoichiometric (two-fold) excess of M.HhaI over 5′-G**C**GC-3′ recognition sequences. This is because the coupling reaction of aziridine cofactor **7** with the target cytosine results in one covalently-linked product, which stays tightly bound to the enzyme (see Section 2.2) and prevents further turnover. With the natural cofactor AdoMet **1**, a transfer reaction occurs, resulting in two products, the methylated DNA and the demethylated cofactor *S*-adenosyl-l-homocysteine, and dissociation of the enzyme from the product complex can readily occur to allow further turnover.

### 2.4. Localization of Streptavidin-Biotin Complexes on Plasmid DNA by Electron Microscopy

**Figure 4:**
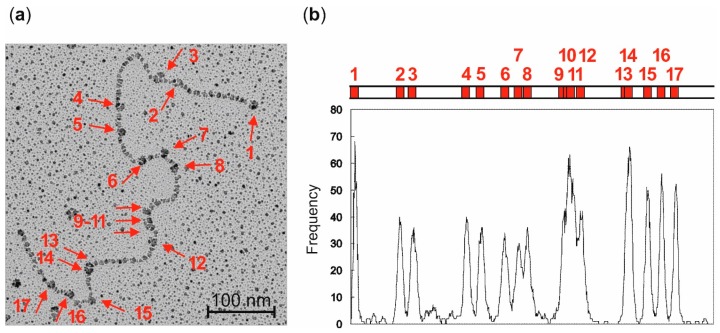
Electron microscopic localization of streptavidin-biotin complexes on labeled LpUC19 plasmid DNA. (**a**) Electron microscopic picture of one LpUC19 plasmid, which was biotinylated with M.HhaI and aziridine cofactor **5** and incubated with streptavidin. The streptavidin molecules are visible as bulges on the plasmid DNA and can be located on all 17 M.HhaI recognition sequences indicated by red arrows. Position numbering corresponds to the schematic representation of LpUC19 plasmid DNA and numbered M.HhaI recognition sequences (red boxes) in (**b**); (**b**) Frequency of bulges on 84 plasmids observed over the entire DNA length. Although all streptavidin molecules can be individually observed in the electron microscopic pictures (see (**a**)), streptavidin molecules bound to positions 9–11 and 13–14 are too close to be resolved in this overlay.

The sequence-specificity of the labeling reaction was directly verified by electron microscopy. LpUC19 plasmid DNA was biotinylated with M.HhaI and the 7-modified aziridine cofactor **5** as described above. The biotinylated plasmid DNA was purified and incubated with streptavidin. The excess of streptavidin was removed by gel filtration and the plasmid prepared for electron microscopy. Using this procedure, only a small amount of streptavidin-crosslinked plasmid molecules was found by electron microscopy. Streptavidin molecules bound to DNA were observed as bulges on LpUC19 plasmid DNA (Figure 4a). Analysis of 84 plasmid molecules with added streptavidin demonstrated a clear preference for the predicted positions of M.HhaI recognition sequences (Figure 4b).

### 2.5. CpG-Methylation Detection

**Figure 5:**
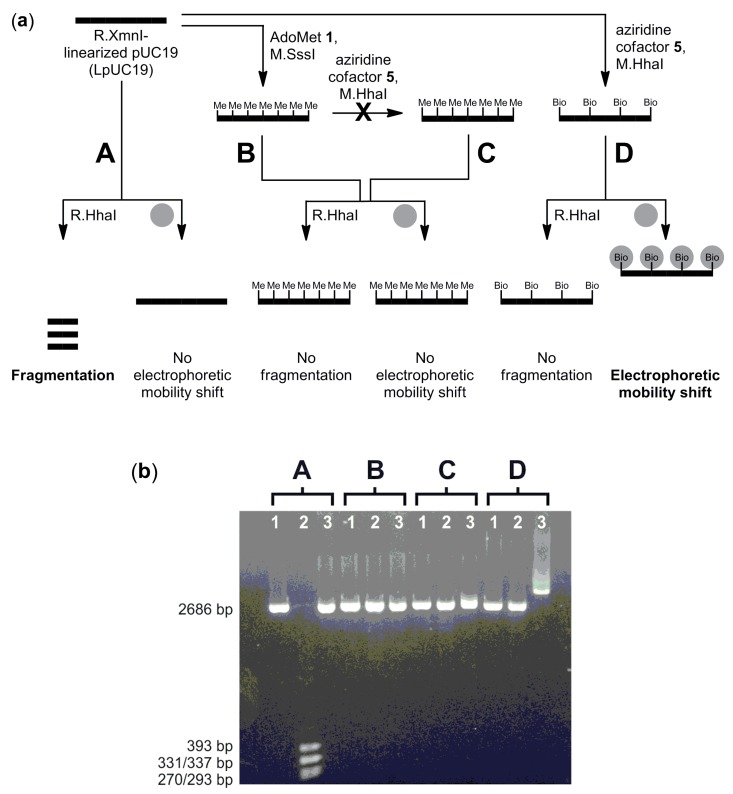
CpG-methylation detection using aziridine cofactor **5** and M.HhaI. (**a**) Reaction scheme to demonstrate specific detection of non-methylated CpG sequences within the M.HhaI recognition sequence by electromobility shift assay (EMSA) (grey sphere = streptavidin; Bio = biotin); (**b**) Agarose gel (1%) electrophoresis of unmodified (A), CpG-methylated (B), CpG-methylated and treated with **5** and M.Hhal (C) and biotin-labeled (D) linearized pUC19 plasmid DNA (LpUC19). Lanes 1: plasmid DNA from A, B, C or D; Lanes 2: plasmid DNA from A, B, C or D incubated with R.Hhal; Lanes 3: plasmid DNA from A, B, C or D incubated with streptavidin. The slight smearing of the DNA in Lane D3 can be explained by a small amount of streptavidin-crosslinked plasmid molecules as observed by electron microscopy. The DNA length is given in base pairs (bp).

DNA biotinylation with aziridine cofactor **5** and M.HhaI was used to specifically detect DNA methylation of LpUC19 plasmid DNA by electromobility shift assay (EMSA) (Figure 5a). Cytosine residues within the 5′-**C**G-3′ DNA sequences (CpG-motifs) were methylated using the DNA cyotsine-C5 MTase from *Spiroplasma* sp. strain MQ1 (M.SssI) and the natural cofactor AdoMet **1**. Non- and CpG-methylated LpUC19 were treated with biotinylated aziridine cofactor **5** and M.HhaI, which can only alkylate the first cytosine residue within the 5′-G**C**GC-3′ DNA sequence if this residue is not blocked by methylation of the inner CpG-motif. The different plasmids were then treated either with the cognate restriction endonuclease R.HhaI, which is blocked by *C*5-alkylation of the first cytosine within the 5′-GCGC-3′ recognition sequence, or streptavidin, leading to fragmentation, no reaction or an electrophoretic mobility shift. Methylation-sensitive enzymatic biotinylation of LpUC19 was analyzed by agarose gel electrophoresis (Figure 5b).

Analysis by restriction endonuclease protection was performed by treating samples of unmodified (A), CpG-methylated (B), CpG-methylated and treated with **5** and M.Hhal (C) or biotin-labeled (D) LpUC19 with R.HhaI. Fragmentation by R.HhaI occurred only with unmodified LpUC19 (A), while CpG-methylated (B or C) or biotinylated (C) LpUC19 was protected against cleavage by R.HhaI (Figure 5b, Lanes 2). These results confirm complete modifications of LpUC19.

For the functional analysis of biotinylation samples of unmodified (A), CpG-methylated (B), CpG-methylated and treated with **5** and M.Hhal (C) or biotin-labeled (D) LpUC19 were incubated with streptavidin. In the presence of streptavidin (Figure 5b, Lanes 3), a reduced electrophoretic mobility caused by the binding of streptavidin was only observed with biotinylated LpUC19 (D), whereas unmodified LpUC19 (A) and CpG-methylated LpUC19 (B or C) did not change their electrophoretic mobility. This result clearly confirms that the labeling reaction with aziridine cofactor **5** and M.HhaI is sequence specific and blocked by CpG methylation. Thus, this system can be used to distinguish between non-methylated and CpG-methylated DNA sequences.

## 3. Experimental Section

Nomenclature: In this section, we have applied the systematic nomenclature of the International Union of Pure and Applied Chemistry (IUPAC) for naming synthesized compounds and assigning NMR data. This is different from the other sections, where the common purine nomenclature is used, e.g., the 7-position in the purine nomenclature translates into the 5-position in the IUPAC nomenclature.

Material: Aziridine cofactor **4** [7], 4-chloro-5-iodo-7*H*-pyrrolo[2,3-*d*]pyrimidine (**6**) [20], 5-*O*-(*t*-butyldimethylsilyl)-2,3-*O*-isopropylidene-α-d-ribofuranosyl chloride (**7**) [17], *N*-(2-propynyl)-2,2,2-trifluoroacetamide (**11**) [21] and aziridine [22,23] were prepared according to literature procedures. (Caution: aziridine is hazardous and should be handled with care). *N*-Hydroxysuccinimidyl biotin (NHS biotin), *tert*-butyldimethylsilyl chloride (TBS-Cl), *N*-ethyldiisopropylamine (EDIA), tetrakis(triphenylphosphine)palladium, triethylamine and trifluoroacetic acid were purchased from Fluka. 4-(Dimethylamino)pyridine (DMAP), methanesulfonyl chloride (MsCl), platinum(IV)oxide, tetrabutylammonium fluoride and tris[2-(2-methoxyethoxy)ethyl]amine (TDA-1) were purchased from Merck. Copper(I) iodide was from Riedel-deHaën. All reagents were of pro analysis (p.a.) grade. Dry solvents were either purchased or dried using common laboratory techniques [24]. The DNA cytosine-C5 MTase M.HhaI was kindly provided by Dr. S. Klimasauskas (Vilnius, Lithuania). Proteinase K was obtained from QIAGEN; DNase (Grade II) was from Roche Applied Science; phosphodiesterases from *Crotalus adamanteus* and from calf spleen were from Sigma; and alkaline phosphatase was from Boehringer. The restriction endonucleases R.HaeII, R.HhaI and R.XmnI were purchased from New England Biolabs; R.TaqI was purchased from MBI Fermentas. Streptavidin was obtained from Gerbu. ODN **I** (5′-TGTCAGCGCATGA-3′) and **II** (5′-TCATGC^Me^GCTGACA-3′; C^Me^ = *C*5-methyl-2′-deoxy–cytidine) were synthesized using a DNA/RNA synthesizer Model 392 from Applied Biosystems and purified by reverse-phase HPLC. pUC19 plasmid DNA was purchased from MBI Fermentas.

General Procedures: All air- or water-sensitive chemical reactions were carried out in dried glassware under an argon atmosphere. Silica gel 60 F_254_ glass plates (Merck, Darmstadt, Germany) were used for TLC. Flash chromatography was carried out using Merck silica gel 60 (40–63 µm). HPLC was performed using a Waters Breeze System equipped with a binary programmable pump system 1525, a dual wavelength absorbance detector 2487 and a Waters inline degasser. NMR spectra were recorded using a Mercury 300 (75 MHz for ^13^C), Inova 400 (400 MHz, 100 MHz and 376 MHz for ^1^H, ^13^C and ^19^F, respectively) and a Unity 500 (500 MHz for ^1^H) (all Varian) in the NMR spectroscopy facility of the institute. CDCl_3_ (δ_H_ = 7.24 and δ_C_ = 77.0) or [D_6_]DMSO (δ_H_ = 2.49 and δ_C_ = 39.5) were used as solvents. Assignments of ^13^C signals are based on ^1^H-, ^13^C-correlated 2D-NMR and on ^13^C-DEPT spectra. Electrospray ionization mass spectra (ESI-MS) were obtained using a Finnigan LCQ DECA XP Plus in the mass spectrometry facility of the institute. Measurements were carried out in the positive ion mode. UV absorption measurements were performed in methanol or water using a Varian Cary 3E spectrometer.

*4-Chloro-5-iodo-7-(5′-O-t-butyldimethylsilyl-2′,3′-O-iso-β-d-propylidene-β-d-ribofuranosyl)-7H-pyrrolo[2,3-d]pyrimidine* (**8**): To a suspension of powdered KOH (280 mg, 5.0 mmol) in dry acetonitrile (15 mL) under argon atmosphere was added tris[2-(2-methoxyethoxy)ethyl]amine (TDA-1) (200 µL, 0.6 mmol) and the mixture stirred at room temperature for 30 min. 4-Chloro-5-iodo-7*H*-pyrrolo[2,3-*d*]pyrimidine (**6**) (500 mg, 1.79 mmol) was added, and the mixture was stirred for 30 min at room temperature. A freshly-prepared solution of 5-*O*-(*t*-butyldimethylsilyl)-2,3-*O*-isopropylidene-α-d-ribofuranosyl chloride (**7**) (3.61 mmol) in THF (8 mL) was added, and the reaction mixture was stirred at room temperature for 2 days. The resulting black suspension was diluted by the addition of ethyl acetate (60 mL) and passed through a paper filter. The solvents were removed under reduced pressure, and the crude product was purified by column chromatography (silica gel, elution with ethyl acetate/hexane 15:85) to give nucleoside **8** (431 mg, 43%) as a light yellow oil (R*_f_* 0.43, ethyl acetate/hexane 20:80). ^1^H-NMR (500 MHz, CDCl_3_): δ = 0.12 (s, 3H, SiCH_3_a), 0.12 (s, 3H, SiCH_3_b), 0.92 (s, 9H, SiC(CH_3_)_3_), 1.38 (s, 3H, isopropylidene-CH_3_a), 1.65 (s, 3H, isopropylidene-CH_3_b), 3.81 (dd, ^2^*J* = 11.29 Hz, ^3^*J* = 3.05 Hz, 1H, H5′a), 3.92 (dd, ^2^*J* = 11.60 Hz, ^3^*J* = 2.75 Hz, 1H, H5′b), 4.40 (q, ^3^*J* = 2.75 Hz, 1H, H4′), 4.90 (dd, ^3^*J* = 2.44 Hz, ^3^*J* = 6.10 Hz, 1H, H3′), 4.94 (dd, ^3^*J* = 3.05 Hz, ^3^*J* = 6.10 Hz, 1H, H2′), 6.43 (d, ^3^*J* = 3.05 Hz, 1H, H1′), 7.78 (s, 1H, H6), 8.65 (s, 1H, H2); ^13^C-NMR (100 MHz, CDCl_3_): δ = −5.38 (SiCH_3_a), −5.22 (SiCH_3_b), 18.43 (SiC(CH_3_)_3_), 25.38 (isopropylidene-CH_3_a), 25.98 (SiC(CH_3_)_3_), 27.32 (isopropylidene-CH_3_b), 52.13 (C5), 63.48 (C5′), 80.79 (C4′), 85.38 (C3′), 86.19 (C2′), 90.77 (C1′), 114.02 (isopropylidene-C(CH_3_)_2_), 117.24 (C9), 131.89 (C6), 150.25 (C2), 150.86 (C8), 152.40 (C4).

*4-Chloro-5-iodo-7-(2′,3′-O-isopropylidene-β-d-ribofuranosyl)-7H-pyrrolo[2,3-d]pyrimidine* (**9**): A solution of nucleoside **8** (348 mg, 0.61 mmol) in THF (10 mL) was cooled to 0 °C. After the addition of tetrabutylammonium fluoride (TBAF) (291 mg, 0.92 mmol), the reaction mixture was stirred at 0 °C for 2 h. The reaction mixture was allowed to warm up to room temperature, and the solvent was removed under reduced pressure. The residue was dissolved in ethyl acetate (10 mL), and the organic layer was washed with water (2 mL) and brine (2 mL). The organic layer was dried over magnesium sulfate, filtered, and the solvent was removed under reduced pressure. Purification by column chromatography (silica gel, elution with ethyl acetate/hexane 40:60) gave nucleoside **9** (192 mg, 69%) as a light yellow foam (R*_f_* 0.23, ethyl acetate/hexane 40:60). ^1^H-NMR (500 MHz, CDCl_3_): δ = 1.37 (s, 3H, isopropylidene-CH_3_a), 1.64 (s, 3H, isopropylidene-CH_3_b), 3.81 (t, ^2^*J* = ^3^*J* = 10.68 Hz, 1H, H5′a), 3.95 (dd, ^2^*J* = 12.51 Hz, ^3^*J* = 1.83 Hz, 1H, H5′b), 4.47–4.49 (m, 1H, H4′), 4.76 (d, ^3^*J* = 10.07 Hz, 1H, 5′-OH), 5.09 (dd, ^3^*J* = 2.14 Hz, ^3^*J* = 6.10 Hz, 1H, H3′), 5.19 (dd, ^3^*J* = 4.88 Hz, ^3^*J* = 6.10 Hz, 1H, H2′), 5.88 (d, ^3^*J* = 4.88 Hz, 1H, H1′), 7.51 (s, 1H, H6), 8.63 (s, 1H, H2); ^13^C-NMR (75 MHz, CDCl_3_): δ = 25.30 (isopropylidene-CH_3_a), 27.54 (isopropylidene-CH_3_b), 51.94 (C5), 63.09 (C5′), 81.16 (C4′), 83.28 (C3′), 85.76 (C2′), 94.58 (C1′), 114.44 (isopropylidene-C(CH_3_)_2_), 118.72 (C9), 134.55 (C6), 149.56 (C2), 150.60 (C8), 153.67 (C4); ESI-MS *m*/*z* (relative intensity): 452.3 (9) [M + H]^+^, 280.5 (100) [4-chloro-5-iodo-7*H*-pyrrolo[2,3-*d*]pyrimidine + H]^+^.

*4-Amino-5-iodo-7-(2′,3′-O-isopropylidene-β-d-ribofuranosyl)-7H-pyrrolo[2,3-d]pyrimidine* (**10**): A solution of nucleoside **9** (981 mg, 2.17 mmol) in methanol (80 mL) was cooled to 0 °C and then saturated with gaseous ammonia. A pre-cooled autoclave was filled with the solution, sealed and heated to 80–85 °C overnight. The autoclave was allowed to cool to room temperature; the solution was removed, and the solvent was evaporated under reduced pressure. The crude product was purified by column chromatography (silica gel, elution with methanol/methylene chloride 5:95) to yield nucleoside **10** (557 mg, 59%) as a light yellow foam (R*_f_* 0.21, methanol/methylene chloride 5:95). ^1^H-NMR (400 MHz, [D_6_]DMSO): δ = 1.31 (s, 3H, isopropylidene-CH_3_a), 1.53 (s, 3H, isopropylidene-CH_3_b), 3.49–3.59 (m, 2H, H5′), 4.10–4.13 (m, 1H, H4′), 4.90 (dd, ^3^*J* = 3.02 Hz, ^3^*J* = 6.32 Hz, 1H, H3′), 5.11 (dd, ^3^*J* = 3.29 Hz, ^3^*J* = 6.31 Hz, 1H, H2′), 5.16 (t, ^3^*J* = 5.49 Hz, 1H, 5′-OH), 6.19 (d, ^3^*J* = 3.30 Hz, 1H, H1′), 6.76 (s, br, 2H, NH_2_), 7.69 (s, 1H, H6), 8.12 (s, 1H, H2); ^13^C-NMR (75 MHz, [D_6_]DMSO): δ = 25.68 (isopropylidene-CH_3_a), 27.59 (isopropylidene-CH_3_b), 52.84 (C5), 62.05 (C5′), 81.43 (C3′), 84.03 (C2′), 85.99 (C4′), 89.28 (C1′), 103.67 (C9), 113.66 (isopropylidene-C(CH_3_)_2_), 127.81 (C6), 150.22 (C8), 152.65 (C2), 157.76 (C4); ESI-MS *m*/*z* (relative intensity): 433.2 (100) [M + H]^+^, 261.5 (8) [4-amino-5-iodo-7*H*-pyrrolo[2,3-*d*]pyrimidine + H]^+^.

*4-Amino-7-(2′,3′-O-isopropylidene-β-d-ribofuranosyl)-5-[1″-(3″-trifluoroacetamido)prop-1″-ynyl]-7H-pyrrolo[2,3-d]pyrimidine* (**12**): To a solution of nucleoside **10** (295 mg, 0.68 mmol) in dry DMF (10 mL) under argon atmosphere were added copper(I) iodide (39 mg, 0.21 mmol), *N*-(2-propynyl)-2,2,2-trifluoroacetamide (**11**) (1.05 g, 6.95 mmol), triethylamine (290 µL, 2.08 mmol) and tetrakis(triphenylphosphine)palladium (118 mg, 0.10 mmol). The yellow reaction mixture was stirred at room temperature overnight. The reaction was quenched by the addition of Dowex^®^ 1 × 8 anion exchange resin (1.3 g, loaded with hydrogen carbonate) and methanol/methylene chloride (1:1 mixture, 15 mL). After stirring at room temperature for 40 min, the mixture was filtered through Celite, and the solvents were removed under reduced pressure. The crude product was purified by column chromatography (silica gel, elution with methanol/methylene chloride 7:93) to give nucleoside **12** (305 mg, 98%) as a light yellow foam (R*_f_* 0.23, methanol/methylene chloride 7:93). ^1^H-NMR (400 MHz, [D_6_]DMSO): δ = 1.31 (s, 3H, isopropylidene-CH_3_a), 1.53 (s, 3H, isopropylidene-CH_3_b), 3.51–3.57 (m, 2H, H5′), 4.13–4.16 (m, 1H, H4′), 4.32 (d, ^3^*J* = 5.22 Hz, 2H, H3″), 4.91 (dd, ^3^*J* = 2.75 Hz, ^3^*J* = 6.04 Hz, 1H, H3′), 5.12 (dd, ^3^*J* = 3.30 Hz, ^3^*J* = 6.05 Hz, 1H, H2′), 5.17, (t, ^3^*J* = 5.36 Hz, 1H, 5′-OH), 6.19 (d, ^3^*J* = 3.30 Hz, 1H, H1′), 7.95 (s, 1H, H6), 8.14 (s, 1H, H2), 10.10 (t, ^3^*J* = 5.22 Hz, 1H, 3″-NH); ^13^C-NMR (100 MHz, [D_6_]DMSO): δ = 25.13 (isopropylidene-CH_3_a), 27.03 (isopropylidene-CH_3_b), 29.88 (C3″), 61.49 (C5′), 75,85 (C2″), 80.91 (C3′), 83.55 (C2′), 85.58 (C4′), 86.89 (C1″), 88.95 (C1′), 94.42 (C5), 102.05 (C9), 112.91 (isopropylidene-C(CH_3_)_2_), 115.62 (q, ^1^*J* = 286 Hz, CF_3_), 126.83 (C6), 149.08 (C8), 152.72 (C2), 156.07 (q, ^2^*J* = 36.4 Hz, COCF_3_), 157.30 (C4); ^19^F-NMR (376 MHz, [D_6_]DMSO): δ = −69.72 (CF_3_); ESI-MS *m*/*z* (relative intensity): 456.4 (12) [M + H]^+^, 284.4 (100) [4-amino-5-[1′-(3′-trifluoroacetamido)prop-1-ynyl)-7*H*-pyrrolo[2,3-*d*]pyrimidine + H]^+^.

*4-Amino-7-(2′,3′-O-isopropylidene-β-d-ribofuranosyl)-5-[1″-(3″-trifluoroacetamido)propyl]-7H-pyrrolo[2,3-d]pyrimidine* (**13**): To a solution of nucleoside **12** (305 mg, 0.67 mmol) in methanol (75 mL) was added platinum(IV)oxide (10 mg, 44 µmol), and hydrogen gas was bubbled through the solution at room temperature for 5 h. The catalyst was removed by filtration through Celite and washed with methanol. The solvent was removed under reduced pressure, and the crude product was purified by column chromatography (silica gel, elution with methanol/methylene chloride 5:95). Nucleoside **13** (248 mg, 81%) was obtained as a light yellow solid (R*_f_* 0.30, methanol/methylene chloride 5:95). ^1^H-NMR (400 MHz, [D_6_]DMSO): δ = 1.31 (s, 3H, isopropylidene-CH_3_a), 1.53 (s, 3H, isopropylidene-CH_3_b), 1.75–1.82 (m, 2H, H2″), 2.75–2.79 (m, 2H, H1″), 3.25–3.30 (m, 2H, H3″), 3.48–3.58 (m, 2H, H5′), 4.06–4.09 (m, 1H, H4′), 4.88 (dd, ^3^*J* = 3.02 Hz, ^3^*J* = 6.32 Hz, 1H, H3′), 5.11 (dd, ^3^*J* = 3.57 Hz, ^3^*J* = 6.32 Hz, 1H, H2′), 5.16 (t, ^3^*J* = 5.64 Hz, 1H, 5′-OH), 6.14 (d, ^3^*J* = 3.84 Hz, 1H, H1′), 6.65 (s, br, 2H, NH_2_), 7.14 (s, 1H, H6), 8.04 (s, 1H, H2), 9.43 (t, ^3^*J* = 5.36 Hz, 3″-NH); ^13^C-NMR (75 MHz, [D_6_]DMSO): δ = 23.53 (C2″), 25.70 (isopropylidene-CH_3_a), 27.62 (isopropylidene-CH_3_b), 29.76 (C1″), 39.26 (C3″), 62.14 (C5′), 81.53 (C3′), 83.62 (C2′), 85.42 (C4′), 89.07 (C1′), 102.46 (C9), 113.70 (isopropylidene-C(CH_3_)_2_), 115.35 (C5), 116.46 (q, ^1^*J* = 286.1 Hz, CF_3_), 120.05 (C6), 150.86 (C8), 151.69 (C2), 156.70 (q, ^2^*J* = 35.7 Hz, COCF_3_), 157.84 (C4); ^19^F-NMR (376 MHz, [D_6_]DMSO): δ = −74.74 (CF_3_); ESI-MS *m*/*z* (relative intensity): 460.4 (88) [M + H]^+^, 288.5 (100) [4-amino-5-[1′-(3′-trifluoroacetamido)propyl]-7*H*-pyrrolo[2,3-*d*]pyrimidine + H]^+^.

*4-Amino-7-(2′,3′-O-isopropylidene-5′-O-mesyl-β-d-ribofuranosyl)-5-[1″-(3″-trifluoroacetamido)propyl]-7H-pyrrolo[2,3-d]pyrimidine* (**14**): To a solution of nucleoside **13** (72 mg, 157 µmol) in dry methylene chloride (7 mL) under argon atmosphere were added dimethylaminopyridine (DMAP) (20 mg, 164 µmol) and triethylamine (66 µL, 474 µmol), and the mixture was cooled to 0 °C in an ice bath. Methanesulfonyl chloride (MsCl) (40 µL, 515 µmol) was slowly added, and the reaction mixture was stirred at 0 °C for 2 h. The reaction was quenched by adding an ice-cold, saturated sodium hydrogen carbonate solution (1 mL), and the organic phase was removed. The aqueous layer was extracted with ice-cold chloroform (3 × 2 mL), and the combined organic layers were dried over magnesium sulfate. After filtration, the solvent was removed under reduced pressure, and the crude product was purified by column chromatography (silica gel, methanol/methylene chloride 7:93) to give nucleoside **14** (40 mg, 46%) as a light yellow solid (R*_f_* 0.47, methanol/methylene chloride 10:90). ^1^H-NMR (400 MHz, [D_6_]DMSO): δ = 1.33 (s, 3H, isopropylidene-CH_3_a), 1.54 (s, 3H, isopropylidene-CH_3_b), 1.77–1.81 (m, 2H, H2″), 2.77 (t, ^3^*J* = 7.42 Hz, 2H, H1″), 3.12 (s, 3H, mesyl-CH_3_), 3.24–3.32 (m, 2H, H3″), 4.31–4.35 (m, 2H, H5′), 4.38–4.42 (m, 1H, H4′), 4.99 (dd, ^3^*J* = 2.75 Hz, ^3^*J* = 6.32 Hz, 1H, H3′), 5.23 (dd, ^3^*J* = 3.02 Hz, ^3^*J* = 6.32 Hz, 1H, H2′), 6.23 (d, ^3^*J* = 3.02 Hz, 1H, H1′), 6.68 (s, br, 2H, NH_2_), 7.13 (s, 1H, H6), 8.07 (s, 1H, H2), 9.42 (t, br, 1H, 3″-NH); ^19^F-NMR (376 MHz, [D_6_]DMSO): δ = −74.74 (CF_3_).

*4-Amino-7-(5′-O-mesyl-β-d-ribofuranosyl)-5-[1″-(3″-trifluoracetamido)propyl)-7H-pyrrolo[2,3-d]pyrimidine* (**15**): A solution of nucleoside **14** (40 mg, 74 µmol) in aqueous trifluoroacetic acid (TFA) (70%, 2 mL) was stirred at room temperature for 45 min. The solvent was removed under reduced pressure and the remaining solvent co-evaporated with ethanol and methylene chloride. The product **15** (R*_f_* 0.22, methanol/methylene chloride 10:90) was directly used in the following reaction.

*4-Amino-7-(5′-N-aziridinyl-5′-deoxy-β-d-ribofuranosyl)-5-[1″-(3″-aminopropyl)-7H-pyrrolo[2,3-d]pyrimidine* (**16**): Crude nucleoside **15** from the previous step was dissolved in a mixture of dry aziridine (1 mL, 16.3 mmol) and EDIA (300 µL, 1.8 mmol) under argon atmosphere, and the reaction mixture was stirred at room temperature for 4 days. The reaction progress was monitored by analytical reverse-phase HPLC (Prontosil-ODS, 5 µm, 120 Å, 250 × 4.6 mm, Bischoff, Leonberg, Germany). Compounds were eluted with acetonitrile (7% for 5 min, followed by linear gradients to 31.5% in 10 min, to 35% in 15 min and to 70% in 5 min) in triethylammonium acetate buffer (0.1 M, pH 7.0) and a flow rate of 1 mL/min. The product **16** eluted with a retention time of 10.2 min (UV detection at 280 nm and 300 nm). Volatile compounds were removed under reduced pressure, and the residue was dissolved in triethylammonium hydrogen carbonate buffer (4 mL, 0.1 M, pH 8.6) to cleave off the trifluoroacetamido group completely. The crude product was purified by preparative reverse-phase HPLC (Prontosil-ODS, 5 µm, 120 Å, 250 × 8 mm, Bischoff). Compounds were eluted with acetonitrile (7% for 5 min, followed by linear gradients to 21% in 15 min and to 70% in 5 min) in triethylammonium hydrogen carbonate buffer (0.01 M, pH 8.6) at a flow rate of 3 mL/min. Fractions containing the product **16** (retention time 16.8 min, UV detection at 280 nm and 310 nm) were combined and stored at −80 °C. The amount of product **16** (10 mg, 39% from **14**) in the combined fractions was determined by UV spectroscopy using the published extinction coefficient ε^279^ = 8500 L·mol^−1^·cm^−1^ of 4-amino-7-(2′-deoxy-β-d-erythro-pentofuranosyl)-5-[1″-(5″-trifluoroacetamido)pentyl]-7*H*-pyrrolo[2,3-*d*]pyrimidine [18].

*4-Amino-(5′-N-aziridinyl-5′-deoxy-β-d-ribofuranosyl)-5-[1″-(N-biotinyl-3″-aminopropyl)]-7H-pyrrolo[2,3-d]pyrimidine* (**5**): To a solution of nucleoside **16** (10 mg, 29 µmol) in triethylammonium hydrogen carbonate buffer (12 mL, 0.01 M, pH 8.6) containing acetonitrile was added *N*-hydroxysuccinimidyl biotin (NHS biotin) (10.2 mg, 30 µmol) in DMSO (500 µL). The reaction was stirred at room temperature for 2 h. The progress of the reaction was monitored by analytical reverse-phase HPLC (Prontosil-ODS, 5 µm, 120 Å, 250 × 4.6 mm, Bischoff). Compounds were eluted with acetonitrile (7% for 5 min, followed by linear gradients to 31.5% in 10 min, to 35% in 15 min and to 70% in 5 min) in triethylammonium acetate buffer (0.1 M, pH 7.0) at a flow rate of 1 mL/min. The product **5** eluted with a retention time of 20.8 min (UV detection at 280 nm and 300 nm). The crude product was purified by preparative reverse-phase HPLC (Prontosil-ODS, 5 µm, 120 Å, 250 × 8 mm, Bischoff). Compounds were eluted with acetonitrile (7% for 5 min, followed by linear gradients to 31.5% in 5 min, to 35% in 10 min and to 70% in 5 min) in triethylammonium hydrogen carbonate buffer (0.01 M, pH 8.6) at a flow rate of 3 mL/min. Fractions containing the product **5** (retention time 14.8 min, UV detection at 280 nm and 300 nm) were combined, and the solvent was removed by lyophilization. The aziridine cofactor **5** (5.2 mg, 32%) was obtained as a white solid. ^1^H-NMR (300 MHz, [D_6_]DMSO): δ = 1.10–1.15 (m, 2H, aliphat. biotin-H), 1.25–1.35 (m, 4H, aziridine-H), 1.45–1.55 (m, 2H, aliphat. biotin-H), 1.55–1.63 (m, 2H, aliphat. biotin-H), 1.64–1.72 (m, 2H, H2″), 2.04–2.06 (m, 2H, aliphat. biotin-H), 2.28–2.34 (m, 1H, H5′a), 2.46–2.50 (m, 1H, H5′b), 2.54–2.58 (m, 1H, biotin-SCH_2_a), 2.72–2.77 (m, 2H, H1″), 2.77–2.83 (m, 1H, biotin-SCH_2_b), 3.05–3.16 (m, 3H, biotin-SCHR, H3″), 3.89–3.94 (m, 1H, H4′), 4.08–4.15 (m, 2H, biotin-SCHRCH, H3′), 4.25–4.34 (m, 2H, biotin-SCH_2_CH, H2′), 6.05 (d, ^3^*J* = 5.69 Hz, 1H, H1′), 6.35 (s, 1H, biotin-NHa), 6.42 (s, 1H, biotin-NHb), 6.53 (s, 2H, NH_2_), 7.10 (s, 1H, H6), 7.79 (t, ^3^*J* = 5.69 Hz, 1H, 3″-NH), 8.03 (s, 1H, H2); ESI-MS: *m*/*z* (relative intensity): 575.25 (100) [M + H]^+^, 418.18 (7) [4-amino-5-[1′-(N-biotinyl-3′-aminopropyl)]-7*H*-pyrrolo[2,3-*d*]pyrimidine + H]^+^.

Biotin labeling of duplex ODN **I·II** with M.HhaI: Molecular extinction coefficients at 260 nm of ODN **I** and **II** were calculated according to the nearest neighbor method [25], and concentrations were determined by UV spectroscopy. Annealing of the complementary strands **I** and **II** was performed by heating an equimolar solution to 95 °C for 2 min followed by slow cooling to room temperature.

A solution (400 µL) of aziridine cofactor **5** (32 nmol, 80 µM), duplex ODN **I·II** (4 nmol, 10 µM) and M.HhaI (4.4 nmol, 11 µM) in buffer (10 mM Tris hydrochloride, pH 7.4, 50 mM sodium chloride, 0.05 mM ethylenediaminetetraacetic acid and 2 mM β-mercaptoethanol) were incubated at 37 °C. The progress of the reaction was monitored by anion exchange HPLC (Poros 10 HQ, 10 µm, 4.6 × 10 mm, Applied Biosystems). Compounds were eluted with aqueous potassium chloride (0.2 M for 5 min, followed by linear gradients to 0.4 M in 5 min, to 0.6 M in 20 min and to 1 M in 5 min) at a flow rate of 4 mL/min. The labeled duplex ODN **I^5^·II** was released from the protein-DNA complex by incubation at 65 °C for 30 min, and complete release was verified by anion exchange chromatography (as described above). Residual aziridine cofactor **5** was removed by gel filtration using a NAP-5 column (Amersham, Biosciences, Freiburg, Germany) following the instructions of the supplier. The presence and functionality of biotin in the duplex ODN **I^5^·II** was verified by the addition of streptavidin (25 µg) to the **I^5^·II** solution (25 µL, 0.25 nmol) and incubation at 37 °C for 30 min. Binding of streptavidin to **I^5^·II** was confirmed by anion exchange HPLC (as described above).

For enzymatic fragmentation, desalted duplex ODN **I^5^·II** (10 nmol) was dried by lyophilization and the residue dissolved in buffer (200 µL, 10 mM potassium phosphate, pH 7.0, 10 mM magnesium chloride). DNase I (7.7 U), phosphodiesterase from *Crotalus adamanteus* (576 mU), phosphodiesterase from calf spleen (768 mU) and alkaline phosphatase (4.8 U) were added and the solution incubated at 37 °C for 24 h. The nucleoside composition of the fragmentation reaction was analyzed by reverse-phase HPLC (Symmetry C-18, 5 µm, 100 Å, 100 × 4.6 mm, Waters, Eschborn, Germany). Compounds were eluted with acetonitrile (linear gradient from 0%–7% in 7 min, followed by linear gradients to 8% in 10 min and to 100% in 13 min) at a flow rate of 1 mL/min. The modified nucleoside dC**^5^** (retention time 22.9 min, detection at 254 nm and 280 nm) was collected, dried by lyophilization and analyzed by mass spectrometry. ESI-MS *m*/*z* (relative intensity): 824.4 (100) [M + Na]^+^.

Biotin labeling of pUC19 plasmid DNA with M.HhaI: Linearization of pUC19 plasmid DNA (10 µg) was carried out by incubation with the restriction endonuclease R.XmnI (100 U) in buffer (40 µL, 10 mM Tris hydrochloride, pH 7.9, 10 mM magnesium chloride, 50 mM sodium chloride, 1 mM 1,4-dithiothreitol and 1 mg/mL bovine serum albumin) at 37 °C for 1 h. The linearized pUC19 (LpUC19) was either directly used for following experiments or stored at 4 °C.

Solutions of LpUC19 (1 µg, 0.56 pmol, 9.6 pmol M.HhaI recognition sites), aziridine cofactor **5** or **4** (80 µM) and M.HhaI (18.5 pmol) in buffer (40 µL, 10 mM Tris hydrochloride, pH 7.4, 50 mM sodium chloride, 0.05 mM ethylenediaminetetraacetic acid and 2 mM β-mercaptoethanol) were incubated at 37 °C. Aliquots (8 µL) were taken after different incubation times and the labeling reaction quenched by heating to 65 °C for 20 min. Each aliquot was supplemented with 10 × NEB2 buffer (1 µL, 100 mM Tris hydrochloride, pH 7.9, 100 mM magnesium chloride, 500 mM sodium chloride, 10 mM 1,4-dithiothreitol, New England Biolabs) and R.HaeII (1 µL, 4 U) and incubated at 37 °C for 1 h. Fragmentation was analyzed by agarose gel (1%) electrophoresis.

Localization of streptavidin-biotin complexes on plasmid DNA by electron microscopy: LpUC19 plasmid DNA was biotinylated using aziridine cofactor **5** and M.HhaI as described above and heated at 65 °C for 20 min. Labeled LpUC19 was isolated using the QIAquick PCR Purification Kit from QIAGEN (Hilden, Germany) and eluted with buffer (50 µL, 10 mM Tris hydrochloride, pH 8.5). Streptavidin (20 µg protein/1 µg plasmid DNA) was added and the solution incubated at room temperature for 1 h. The excess of streptavidin was removed by gel filtration on sepharose CL-4B (Pharmacia, Uppsala, Sweden). The DNA was diluted in buffer (10 mM triethylammonium hydrochloride, pH 7.9, 10 mM magnesium chloride) to a final concentration of 1 ng/µL and directly absorbed on a glimmer surface [26]. Positions of the streptavidin-biotin complexes of 84 plasmids were determined from electron micrographs taken statistically with an EM400 (Philips, Eindhoven, The Netherlands). Contour lengths were measured with a LM4 (Brühl, Nürnberg, Germany) and the data analyzed with a computer program [27].

CpG-methylation detection: CpG-methylation of LpUC19 (6 µg) was carried out with M.SssI (54 pmol) and AdoMet **1** (160 µM) in buffer (40 µL, 10 mM Tris hydrochloride, pH 7.9, 6 mM magnesium chloride, 50 mM sodium chloride, 1 mM 1,4-dithiothreitol and 0.6 mg/mL bovine serum albumin) at 37 °C for 1 h. The reaction was stopped by heating to 65 °C for 20 min. Methylated LpUC19 was purified using the QIAquick PCR Purification Kit (QIAGEN GmbH) according to the instructions of the supplier and eluted with buffer (10 mM Tris hydrochloride, pH 8.5).

Unmodified or CpG-methylated LpUC19 (2 µg, 1.12 pmol, 19.2 pmol M.HhaI recognition sites), aziridine cofactor **5** (80 µM) and M.HhaI (38 pmol) in buffer (100 µL, 10 mM Tris hydrochloride, pH 7.4, 50 mM sodium chloride, 0.05 mM ethylenediaminetetraacetic acid and 2 mM β-mercaptoethanol) were incubated at 37 °C for 2 h and then at 65 °C for 20 min. Plasmid DNA was purified using the QIAquick PCR Purification Kit (QIAGEN GmbH) and eluted with buffer (10 mM Tris hydrochloride, pH 8.5).

Unmodified, CpG-methylated or biotinylated LpUC19 (200 ng) in buffer (10 µL, 10 mM Tris hydrochloride, pH 7.9, 10 mM magnesium chloride, 50 mM sodium chloride, 1 mM 1,4-dithiothreitol and 1 mg/mL bovine serum albumin) were incubated with R.HhaI (2 U) at 37 °C for 1 h. DNA fragmentation was analyzed by agarose gel (1%) electrophoresis.

Unmodified, CpG-methylated or biotinylated LpUC19 (200 ng) in buffer (10 µL, 10 mM Tris hydrochloride, pH 8.5) were incubated with streptavidin (5 µg) at 37 °C for 1 h, and binding of streptavidin was analyzed by the electromobility shift assay (EMSA) using agarose gel (1%) electrophoresis.

## 4. Conclusions

We designed and synthesized a new 7-deazaadenosylaziridine nucleoside **5** with biotin attached to the 7-position and demonstrate that **5** functions as a cofactor for the DNA MTase M.HhaI. The quantitative coupling reaction is sequence specific, as demonstrated by electron microscopy, and biotin is functionally attached to DNA, as evidenced by streptavidin binding to the modified DNA. Several other cofactor analogues have been prepared and used to modify DNA with M.HhaI. They include *N*-adenosylaziridine with azide at the 8-position [8], *N*-adenosyl mustards (which are thought to form reactive aziridinium intermediates) with an azide or terminal alkyne group attached via short linkers to the 8- [12] or *N*6-position [13], as well as AdoMet analogues with extended methyl group replacements for transfer of amino [28,29], terminal alkyne or azide groups [30,31]. These cofactor analogues have in common that unique functional groups, like primary amines, terminal alkynes or azides, are attached to the DNA and a second chemical step is required for DNA labeling. Although this offers flexibility with regard to the reporter groups, it has the disadvantage that the second step can be difficult to follow, especially when labeling long plasmid DNA. This can result in partially-labeled DNA. The 7-modified aziridine cofactor **5** already contains biotin as the reporter group, and DNA labeling is achieved in one step, which can be easily analyzed with suitable restriction endonucleases.

It is interesting to note that some of these cofactor analogues for two-step DNA labeling contain substituents at the 8-position. Apparently, the steric demand of these substituents, like azide, propargylamine or 4-azidobutylamine, is smaller than the *N*-(4-aminobutyl)biotinamide in the 8-modified *N*-adenosylaziridine **4**, which is not accepted by M.HhaI (Figure 3b, Lanes 9–12).

The new 7-modified cofactor **5** should also be a substrate for other DNA MTases. In fact, parallel experiments with the adenine-specific DNA MTase M.TaqI show that the 7-modified cofactor **5** is coupled with DNA even more rapidly than the 8-modified cofactor **4**. In addition, three-dimensional structures of further DNA MTases in complex with AdoMet **1** or adenosine derivatives indicate that they should be able to bind either the 7- or the 8-modified aziridine cofactors **5** and **4** for coupling with their DNA recognition sequences [5].

One interesting application of M.HhaI in combination with AdoMet analogues for DNA labeling could be DNA methylation detection in mammalian DNA. Using M.HhaI and the aziridine cofactor **5** non-methylated CpG sites within the 5′-G**CG**C-3′ recognition sequence can be labeled with biotin for affinity enrichment and DNA sequencing, while CpG-methylated recognition sequences will be protected against biotinylation. Such an approach has been recently reported for the M.SssI DNA MTase and AdoMet analogues with extended methyl group replacements [31]. It is also feasible that M.HhaI will be capable of coupling other 7-modified aziridine cofactors carrying fluorophores with DNA and use them for CpG-methylation-dependent DNA labeling and detection by DNA mapping [29,32,33]. Thus, SMILing DNA could not only become an interesting tool for sequence-specific labeling of DNA, but also for the exploration of DNA methylation in mammalian cells.
